# Chemical and Colloidal Stability of Carboxylated Core-Shell Magnetite Nanoparticles Designed for Biomedical Applications

**DOI:** 10.3390/ijms140714550

**Published:** 2013-07-12

**Authors:** Márta Szekeres, Ildikó Y. Tóth, Erzsébet Illés, Angéla Hajdú, István Zupkó, Katalin Farkas, Gábor Oszlánczi, László Tiszlavicz, Etelka Tombácz

**Affiliations:** 1 Department of Physical Chemistry and Materials Science, University of Szeged, Aradi Vt. 1, H-6720 Szeged, Hungary; E-Mails: ildiko.toth@chem.u-szeged.hu (I.Y.T.); illes.erzsebet@chem.u-szeged.hu (E.I.); 2 Laboratory of Nanochemistry, Department of Biophysics and Radiation Biology, Semmelweis University, H-1089 Budapest, Nagyvárad tér 4, Hungary; E-Mail: hajdu.angela@med.semmelweis-univ.hu; 3 Department of Pharmacodynamics and Biopharmacy, University of Szeged, Eötvös u. 1, H-6720 Szeged, Hungary; E-Mail: zupko@pharm.u-szeged.hu; 4 Department of Laboratory Medicine, University of Szeged, Semmelweis u. 6, H-6720 Szeged, Hungary; E-Mail: lokine.farkas.katalin@med.u-szeged.hu; 5 Department of Public Health, University of Szeged, Dóm tér 10, H-6720 Szeged, Hungary; E-Mail: oszlanczi.gabor@gmail.com; 6 Department of Pathology, University of Szeged, Állomás u. 2, H-6720 Szeged, Hungary; E-Mail: tiszlats@yahoo.com

**Keywords:** carboxylated magnetite nanoparticles, core-shell nanoparticles, adsorption, surface complexation, chemical stability, iron dissolution, colloidal stability, particle charge, biocompatibility, biomedical application

## Abstract

Despite the large efforts to prepare super paramagnetic iron oxide nanoparticles (MNPs) for biomedical applications, the number of FDA or EMA approved formulations is few. It is not known commonly that the approved formulations in many instances have already been withdrawn or discontinued by the producers; at present, hardly any approved formulations are produced and marketed. Literature survey reveals that there is a lack for a commonly accepted physicochemical practice in designing and qualifying formulations before they enter *in vitro* and *in vivo* biological testing. Such a standard procedure would exclude inadequate formulations from clinical trials thus improving their outcome. Here we present a straightforward route to assess eligibility of carboxylated MNPs for biomedical tests applied for a series of our core-shell products, *i.e*., citric acid, gallic acid, poly(acrylic acid) and poly(acrylic acid-*co*-maleic acid) coated MNPs. The discussion is based on physicochemical studies (carboxylate adsorption/desorption, FTIR-ATR, iron dissolution, zeta potential, particle size, coagulation kinetics and magnetization measurements) and involves *in vitro* and *in vivo* tests. Our procedure can serve as an example to construct adequate physico-chemical selection strategies for preparation of other types of core-shell nanoparticles as well.

## 1. Introduction

Nanobiotechnology can be applied to produce nanosized and/or nanostructured drug formulations for diagnostics, therapy or theranostics. The state-of-the-art of nanobiotechnology is promising [1–8]; but there is a gap between the ideas and the realizations [9]. Basic nanoscience research could not be converted into clinically verified and marketed medical products at a considerable scale as it is shown in a recent review by Etheridge *et al.* [10]. This is especially true of iron oxide nanoparticles (superparamagnetic iron oxide—SPIO, ultra small superparamagnetic iron oxide—USPIO or magnetite nanoparticles—MNPs), for which 66 clinical trials have been initiated [10], but only a few approved formulations can be found in databases such as FDA (U S Food and Drug Administration) or EMA (European Medicines Agency). In addition, the most of them have subsequently been discontinued or withdrawn by the producers. Some examples are Ferumoxides (Feridex I.V., Endorem) [11], Ferumoxsil [12] and Ferumoxtran-10 (Sinerem—Withdrawn from EMA [13]; Combidex—Not yet approved by FDA) [14]). We could identify only one approved and used formulation, Ferumoxytol (Feraheme), but it is approved for treating iron deficiency and thus, iron release from the nanoparticles is expected a priori [15]. One commonly referred formulation VSOP C-184 completed only the first phase clinical trial (2006) and another one, Ferucarbotran (Resovist), the third phase (2003); these are not yet approved [16,17]. Etheridge *et al.* [10] noted that “…academic, government, and industry experts did not expect to see “nanomachines” capable of theranostics (combined therapy and diagnostics) in human beings until 2025”. One of the many reasons for the inefficient progress in medical implementation can be connected with the absence of an appropriate physicochemical testing of the particles. The majority of research studies do not even provide experimental evidence whether an optimization process has been used in designing the given composition. Adequate physicochemical qualification of both the formulation procedure and the end products is inevitable for improving nanoparticle performance and reproducibility. Dufort and co-authors [18] attempted to specify the types of *in vitro* nanoparticle evaluation methods that should be performed prior to *in vivo* tests and highlighted the importance of physicochemical experiments in predicting the fate of nanoparticles *in vivo*. However, the latter had not been classified as a separate group of the *in vitro* experiments.

The procedure of core-shell nanoparticle preparation affects the biological performance of the end-products. The preparation method of the naked MNPs determines their physical properties such as particle size, shape and magnetization [19]. As it is demonstrated, for example, in the work of Rinaldi and co-authors [20], covalent anchoring in many instances can enhance colloidal stability as compared with the physico-chemical adsorption of coating molecules. If a chemical surface modification method is used, however, all the applied chemicals and byproducts should be removed in order to reduce the chemical hazard of the formulation. MNPs designed for cellular uptake should be coated by a negatively charged shell and an uncharged shell is needed for avoiding cellular uptake [21]. Specific labeling or drug-loading of core-shell MNPs can alter the particle-particle interactions *i.e*., affect dispersion stability, which should also be considered during preparation.

In the present paper, we attempt to set up a useful methodology of physicochemical testing of core-shell magnetite nanoparticles. Since the different procedures of core-shell MNP preparation require specific methodologies, we discuss only the process of coating via adsorption of small and macromolecular carboxylates and rely on our own experience [22–26].

Independent of their specific biological functionality, all the MNP formulations must retain their chemical and colloidal stability *in vivo* under the applied magnetic field. It is known that the naked MNPs are both chemically and colloidally unstable in biological fluids even in the absence of magnetic field. Coating shells of proper structure can render them chemically stable via passivation of the magnetic core [27] and provide enhanced colloidal stability by way of increasing electrostatic and/or steric repulsion between the MNPs. The optimization of the carboxylated coatings on magnetite nanoparticles presented in this paper can be adapted to prepare other types of coated nanoparticles as well.

Our protocol of coated MNP preparation eligible for *in vitro* and *in vivo* experiments consists of six basic steps of measuring (i) carboxylate adsorption; (ii) dilution resistance of the adsorbed layer; (iii) iron dissolution; (iv) electrokinetic potential; (v) particle size; and (vi) salt tolerance. We demonstrate the procedure through the examples of citric acid, gallic acid, poly(acrylic acid), and poly(acrylic acid-*co*-maleic acid) coated MNPs (CA@MNP, GA@MNP, PAA@MNP and PAM@MNP, respectively) [22–26]. The results of coating the MNPs with GA, PAA and PAM are published in detail and new experiments for CA-coating are added to the discussion here because of the definite popularity of citric acid as MNP surface modifier in both literature and clinical trials. The experiments listed above give a comprehensive colloidal characterization of carboxylate@MNPs. We note that iron dissolution experiments are inevitable in this case to prove the chemical stability of the MNP core. It is well known that the presence of dissolved ferric ion can cause oxidative stress [28], and it has been also shown that dissolved iron leads to cellular damage [29]. The performance of our optimized carboxylate@MNPs was tested *in vitro* in cytotoxicity, blood sedimentation and blood aggregation experiments, and *in vivo* after intravenous (IV) injection in rat tail. The dissolution and distribution of particles in the cellular environment and rat tail tissue are shown in this paper.

## 2. Results and Discussion

### 2.1. The Strength of Core-Shell Binding

The strength of core-shell binding determines the long-term persistency of nanocomposites. We have measured adsorption and desorption isotherms to evaluate binding affinity and dilution resistance of the coatings. In addition, FTIR-ATR spectra were taken to identify the bonds of the carboxylate and alcohol moieties of the carboxylates to the MNP surface.

#### 2.1.1. Adsorption and Desorption of the Carboxylates

**Figure 1:**
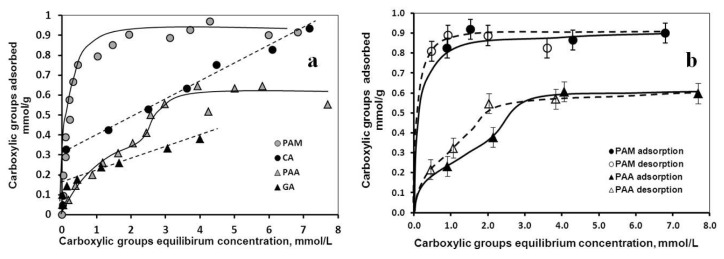
Adsorption isotherms of CA, GA, PAA, and PAM (**a**) and adsorption (**─**) and desorption (----) isotherms of PAA (Δ) and PAM (o) (**b**) on MNP measured at pH ~6 and *I* = 0.01 M. The lines are drawn to guide the eyes. The error bars in (**a**) are omitted for clarity.

The results of the adsorption and desorption measurements performed at pH ~6 and at I = 0.01 M are seen in Figure 1.

The pH and ionic strength were chosen so as to optimize the electrostatic conditions for the adsorption interaction. As it is discussed in detail [23,24], the carboxylates are negatively and the MNPs positively charged at the selected values of pH and ionic strength, with maximized electrostatic attraction between them; a shift in the pH or an increase in the ionic strength would decrease the attraction. The molar amounts of carboxylate groups were chosen to calculate the adsorption/desorption isotherms, because they have primary importance in the electrostatic stabilization. The main features of the isotherms can be best discussed using the Giles classification [30]. H (high-affinity)-type isotherms were obtained for CA, GA and PAM, and L (Langmuir)-type isotherm for PAA. The high-affinity limit was ~0.3 mmol/g for CA and PAM, ~0.15 mmol/g for GA, and 0 mmol/g (*i.e.*, there was no high affinity observed) for PAA. The high affinity limits express the amounts of carboxylates bound practically irreversibly (with an extremely high residence time in the adsorbed layer and a negligible concentration in the dispersion medium). Based on these values, the preference for coating shell formation decreases in the order CA ~ PAM > GA > PAA. The maximum adsorbed amounts of the carboxylates seen as the plateau values of the isotherms vary significantly. Definite values of ~0.9 mmol/g and ~0.6 mmol/g are revealed for PAM and PAA. For CA and GA, however, plateau regions cannot be identified. Instead, a linearly increasing part is obtained in both cases, not characteristic of common adsorption isotherms. These isotherms represent the combined process of adsorption and surface polymerization [25,26]. Regarding the maximum of adsorption, PAM is more preferable for shell formation than PAA, because the higher adsorbed amount of carboxylates implies a thicker coating shell. The much lower equilibrium carboxylate concentration at the plateau of PAM adsorption (~1 mmol/L for PAM *versus* ~4 mmol/L for PAA) also makes it preferable. The amount of free carboxylates in the medium of MNP formulations should be minimal, because of their possible undesirable effects; this is best achieved by keeping their concentration as low as possible during the preparation. In Figure 1b, the isotherms of desorption are plotted together with that of adsorption. The desorption curves run above the adsorption ones for both PAM and PAA suggesting that both coatings are in principle dilution resistant within the time frame of our experiment. We did not attempt measuring desorption of CA and GA because their adsorption is superimposed by surface polymerization making desorption experiments irrelevant. It has been shown for a series of core-shell MNPs [31,32] that shells can be displaced by adsorption of phosphate from biological buffers depending on the size and the anchoring type of coating molecule and the density of coating. Samples resisting shell detachment in aqueous NaCl solutions should be subject to tests in PBS and different cell culture media in further *in vitro* studies.

The adsorption of small molecular or polymeric carboxylates is a widely applied method of core-shell MNP preparation, but the isotherms are rarely measured and analyzed. However, the information extracted from these is inevitable for synthesis design. On the basis of the above discussed isotherm characteristics and parameters, the candidates for coating MNPs can be qualified with respect to the bond strength, dilution resistance and shell compactness. Therefore, high-affinity character, high adsorption density and low equilibrium concentration are expected as the main selection criteria.

#### 2.1.2. Molecular Mechanism of the Carboxylate-MNP Surface Interactions

**Figure 2:**
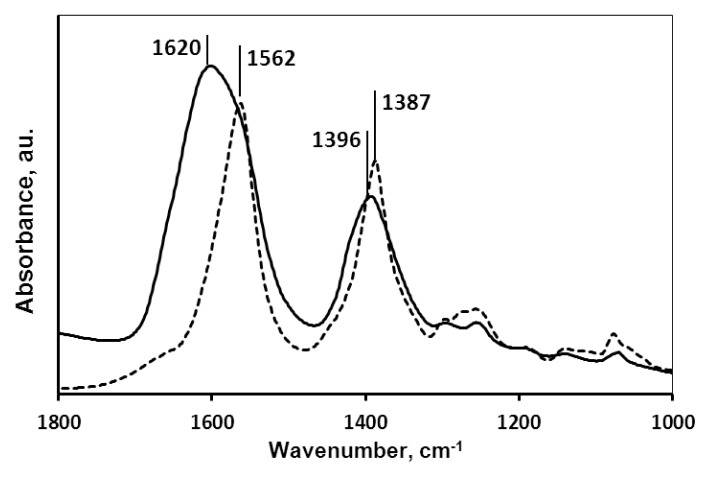
FTIR-ATR spectra of pure CA (---) and CA@MNP at 0.25 mmol/g added amount of CA (─); pH ~6 and *I* = 0.01 M.

Core-shell binding quality was tested by surface spectroscopy. The shifts in the characteristic IR frequencies of the –COOH groups were used to detect the type of binding to the MNP surface. The absorption spectra of GA, PAA and PAM coated MNP samples have been published previously [23,24,26] and that of CA@MNP and pure CA are seen in Figure 2. Due to CA adsorption, the symmetric and asymmetric stretching vibrations of the –COO^−^ groups (dissociated carboxylates, at the pH of the formulation) shifted from 1387 cm^−1^ and 1562 cm^−1^ (pure CA) to 1396 cm^−1^ and 1620 cm^−1^ (CA@MNP) with Δν_sym,COO−_ = 9 cm^−1^ and Δν_asym,COO−_ = 58 cm^−1^, respectively. This indicates inner sphere Fe-carboxylate complex formation. The same was observed for PAM adsorption with frequency changes of Δν_sym,COO−_ = 4 cm^−1^ and Δν_asym,COO−_ = 7 cm^−1^. On the contrary, carboxylate frequencies did not shift to higher values in PAA and GA adsorption; in fact the ν_sym,COO−_ of GA even shifted to lower frequency (Δν_sym,COO−_ = −11 cm^−1^). These results imply that the –COO^−^ groups are not involved in the adsorption of PAA and GA. H-bonding between ≡Fe–OH and non-dissociated –COOH groups (*i.e*., outer-sphere surface complex formation) was identified as the main adsorption mechanism for PAA and direct Fe–O–C– bonding with phenolic OH– groups (*i.e.*, inner-sphere surface complex formation) for GA adsorption.

FTIR-ATR is an adequate surface analysis tool to identify surface bonds; however, it is very important to guarantee that the coating molecules do not interact with the ATR crystal surface itself. For example, on zinc selenide (ZnSe) crystal, citric acid is capable of Zn^2+^ chelation [33] involving the carboxylates and hydroxyls the same way as in the formation of Fe-citrate complexes. We have applied the diamond ATR crystal to exclude the interaction of the carboxylic acids with the crystal surface. Our FTIR-ATR results confirmed that the high-affinity adsorption (a kind of chemical adsorption mechanism) of CA, GA and PAM on MNPs at pH ~6 and I = 0.01 is due to inner-sphere surface complex formation, while the non-high-affinity adsorption (a transition to non-specific physical adsorption mechanism) of PAA is owing to outer-sphere binding. Surface spectroscopy has made it clear that CA, GA and PAM are preferable for MNP coating, while PAA is not.

### 2.2. Chemical Stability of the Core-Shell MNPs

**Figure 3:**
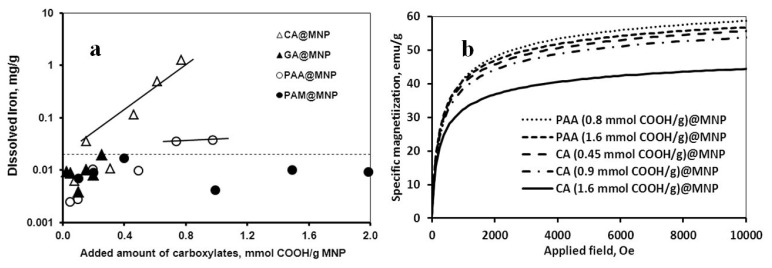
(**a**) Dissolved iron concentration in carboxylate@MNP dispersions (10 g/L in 0.01 M NaCl solution at pH ~6) in the function of the amount of added carboxylates, CA, GA, PAA and PAM. The lines are drawn to CA and PAA data to guide the eyes. The error bars are omitted for the sake of clarity. The dotted line indicates the iron detection limit; (**b**) Specific magnetization curves measured in the dispersions of CA and PAA coated MNPs (100 g/L in water at pH ~6) at different added amounts of the carboxylates.

Chemical stability of the coated nanoparticles was tested in iron dissolution experiments by ICP determination of dissolved iron in the aqueous dispersions of carboxylate@MNPs in 0.01 M NaCl solution at pH ~6. As it is seen in Figure 3a, significant iron dissolution is detected in the presence of CA coating as compared to the other three carboxylate coatings. The maximum measured value of iron dissolution from CA@MNPs was 1.25 mg/g MNP. Rodríguez *et al*. [34] have found similar iron dissolution rate (~1.5 mg/g Fe_3_O_4_) at somewhat different experimental conditions: The specific surface area of their Fe_3_O_4_ was 26 m^2^/g (100 m^2^/g of our MNPs); the dispersion concentration was 0.15 g/L (10 g/L in our experiments); the concentration of CA was 67 mmol/g Fe_3_O_4_ (0.257 mmol/g MNP in our experiments). The above data reflect the known effect of particle size on solubility (Ostwald ripening phenomenon [35]). The smaller the particle size in the colloidal range, the larger the surface curvature and surface free energy are, which in turn promote particle dissolution. It follows that similar degree of iron dissolution can also be achieved at much lower CA concentrations from nanoparticles than from micron-sized particles (0.257 and 67 mmol/g, respectively). We should note that citric acid is also capable of reductive dissolution of iron oxide [36–40], and this corrosion further enhances the rate of nanoparticle decomposition. The latter effect is larger for the higher-surface-area nanoparticles. In contrast to the effect of CA, PAA addition increased iron dissolution only to a small degree (slightly above the detection limit of the experiment): The amount of ~0.035 mg Fe/g MNP is two orders of magnitude smaller than that for CA coating. In the case of GA and PAM coatings, the dissolved iron concentration remained below the detection limit even at the highest added amounts of the carboxylates meaning that the latter coatings passivized the magnetic core of MNPs.

In addition, magnetization curves of the CA@MNP and PAA@MNP dispersions were measured to test the effect of iron dissolution on the core size and magnetization of the MNPs. The results are shown in Figure 3b for some samples. Because iron dissolution leads to the decrease in particle size and the value of saturation magnetization (M_S_) depends on the size of the magnetic core [41–45], the saturation magnetization values are in turn sensitive to iron dissolution (for MNPs from the same lot). As it is seen in Figure 3b, only CA could induce considerable decrease in the M_S_ value. PAA coating (0.8 and 1.6 mmol COOH/g MNP) as well as the lower amounts of CA (0.45 and 0.9 mmol COOH/g MNP) did not alter appreciably the specific magnetization, measured as 53–58 emu/g for all of them. Further increase in the added amount of CA to 1.6 mmol COOH/g MNP lowered the saturation magnetization to M_S_ ~42 emu/g. The latter value corresponds to the magnetic core size of ~5.58 nm as calculated by using the equation for particle size dependence of M_S_[44] known as the “law of approach to saturation”. Haddad and co-workers [43] arrived at similar results by measuring core sizes and magnetization curves in parallel; the diameters of their MNP samples were ~5.5 and ~8.3 nm and the corresponding M_S_ values ~38 and ~50 emu/g, respectively. We calculated from the iron dissolution experiments (Figure 3a) that addition of CA at 1.6 mmol COOH/g MNP concentration reduced the particle size to ~7.2 nm from the original 8 nm (Experimental Section). At ~1.6 mmol COOH/g CA addition the extrapolated value of Fe dissolution is ~30 mg Fe/g and the loss of Fe_3_O_4_ is ~42 mg/g. For particle size calculation we used the value of magnetite density, *ρ* = 5.046 g/cm^3^[46]. Due to the effect of surface spin disorder [45,47], the size of the magnetic mass of MNPs is ~2 nm smaller than the physical particle size (a ~1 nm thick interfacial layer of the particles has an altered magnetic moment or cannot be magnetized at all); thus, the size of the effective magnetic core is ~5.2 nm. This result is close to the above values of 5.58 nm (calculated for ~42 emu/g using the “law of approach to saturation”) and 5.5 nm (at ~38 emu/g measured by Haddad *et al*. [43]).

The results of the iron dissolution supported by magnetization studies show clearly that the CA coated MNPs are not stable chemically. Although the high-affinity adsorption (Figure 1a) and the formation of inner-sphere surface Fe-carboxylate complexes (FTIR-ATR results) imply long-time residence of CA in the coating layer of MNP, we have proven that in parallel with CA adsorption, significant particle degradation/corrosion occurs as well. The presence of dissolved iron in the solution phase of the CA@MNP dispersions as either Fe-citrate complexes or iron hydrolysis products is unacceptable for biomedical applications. Soenen and co-workers [28,29,48] studied the extent of iron dissolution from citrate-, dextran-, carboxydextran-, and lipid-coated MNPs (VSOP C200, Resovist, Endorem, and magnetoliposomes, respectively) in cellular media *in vitro* and found that the citrate-coated VSOP degrades most rapidly and magnetoliposomes possess the highest chemical stability. A range of adverse effects on cell functionality was observed. In addition, MNP degradation inhibited their use for magnetic imaging or targeting at the concentrations not yet toxic for cells, because the magnetic response decreased with decreasing magnetic core size. Their findings underline the absolute necessity of efficient passivation of the MNPs by the coating shell for biomedical application. Our dissolution experiments show that passivation of the PAA-coated nanoparticles is also insufficient, since iron was dissolved to some degree. At the same time, the magnetization measurements reveal that deterioration of particles affects the value of saturation magnetization only at high levels of dissolution, but not at lower. The loss of Fe_3_O_4_ from CA@MNP at 1.6 mmol COOH/g MNP is ~4.2%, while it is not measurable for the PAA-coated MNPs. Thus, the adsorption and iron dissolution tests disqualify CA and PAA and only GA and PAM remain as applicable in biomedical carboxylate@MNP formulations.

### 2.3. Colloidal Stability of the Core-Shell MNPs

The measurements of electrokinetic potential and hydrodynamic diameter of core-shell MNPs are extremely helpful in optimizing the loading of coating molecules (at the given pH and ionic strength, see Section 2.1.1.), and assessing the salt tolerance of the composites at physiological pH (e.g., pH ~7 in the blood). The results are published entirely for PAA and PAM coatings in [23] and [24]. In this paper, we complete the previous partial discussions on CA coating [22,25,26] and compare the characteristic points of all four carboxylated MNPs.

**Figure 4:**
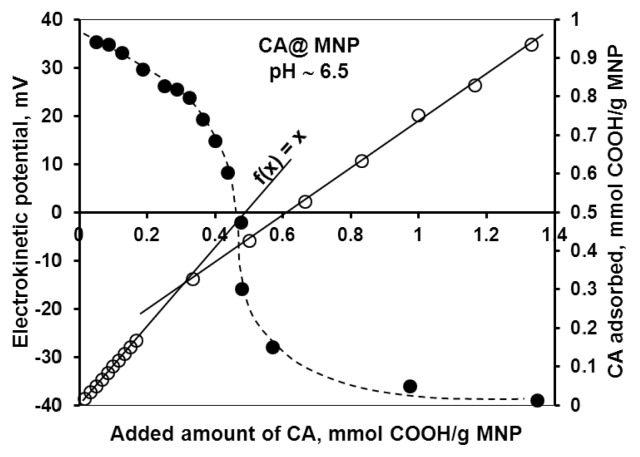
Plots of the electrokinetic potential of CA@MNPs (---) and the adsorbed amount of COOH groups at the MNP surface (─) as a function of COOH addition to MNP dispersion at pH ~6.5 and *I* = 0.01 M. The error bars are omitted for the sake of clarity. The lines are drawn to guide the eyes, except for *f*(*x*) = *x*.

The change in the particle charge due to CA adsorption measured as electrokinetic potential (ζ) of MNP is shown in Figure 4 in parallel with the adsorption data. From the combined adsorption/electrokinetic potential plots, a more detailed picture of adsorption mechanisms can be deduced, which in turn can be used to support the findings of the adsorption and chemical stability studies as well. The same combined plots for PAA and PAM systems are shown in the previous publications [23,24]. Three characteristic COOH concentration values can be identified in Figure 4, also given in Table 1 for all carboxylates. The high-affinity adsorption limit can be defined very precisely with the help of the *f*(*x*) = *x* line drawn to the adsorbed amount *vs.* added amount data points. The *f*(*x*) = *x* line represents practically complete adsorption of the added COOH groups. Adsorption data start to deviate from this line approximately at the high-affinity adsorption limits 0.33, 0.17 and 0.38 mmol COOH/g MNP for CA, GA and PAM, respectively. For PAA adsorption (with non-high-affinity adsorption isotherm), the data points lie below the *f*(*x*) = *x* line in the whole range. The isoelectric point (IEP, the zero value of electrokinetic potential; here the surface charge neutralization point due to adsorption of oppositely charged ions) is found well within the range of high-affinity adsorption for GA and PAM (at 0.05 and 0.17 mmol COOH/g MNP, respectively), but far beyond that for CA (at 0.45 mmol COOH/g MNP). In high-affinity adsorption, the specific interactions overcome electrostatic repulsion. This explains the continuation of adsorption beyond IEP regardless of the like-charge character both of carboxylate ions and of the carboxylated surface. In the CA@MNP system, however, the IEP is not reached at the high-affinity limit, but only at a significantly higher carboxylate concentration. It is clear that not only the adsorption of CA proceeds. The excess amount of COOH above its adsorbed amount is most likely consumed in iron dissolution, as discussed above. The complete overcharging of the MNPs due to carboxylate coverage (ζ ~ (−40) mV) can be seen in the electrokinetic potential plots at around 0.4 mmol COOH/g MNP for all three (GA, PAA and PAM) carboxylates that did not (or only weakly) dissolve iron oxide. However, in the case of CA coating, the advanced dissolution of MNPs increases the characteristic value of complete overcharging to twice the amount for other carboxylates (~0.8 mmol COOH/g MNP).

**Figure 5:**
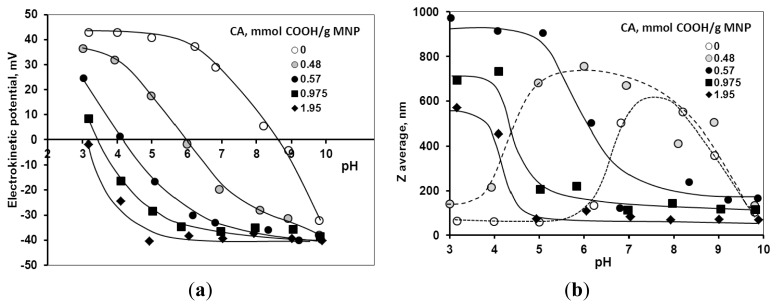
pH-dependent electrokinetic potential (**a**) and particle diameter (**b**) of magnetite nanoparticles at citric acid (CA) loadings of 0, 0.48, 0.57, 0.975 and 1.95 mmol/g. The error bars are omitted for the sake of clarity. The lines are drawn to guide the eyes.

The pH-dependence of electrokinetic potential and hydrodynamic diameter values of the coated MNPs at different citrate loadings is shown in Figure 5. With increasing CA amount, the IEP shifts to lower pH values, and the pH range of highly negatively charged CA@MNPs becomes gradually broader (Figure 5a) similarly to that experienced in all other carboxylic acid formulations [23–26]. Despite their high negative electrokinetic potential at pH >5, all the CA@MNP samples (except for the highest CA loading of 1.95 mmol COOH/g MNP) undergo aggregation (Figure 5b). The aggregation at high electrokinetic potential values can be caused by dissolved ferric ions or iron-citrate complexes.

**Figure 6:**
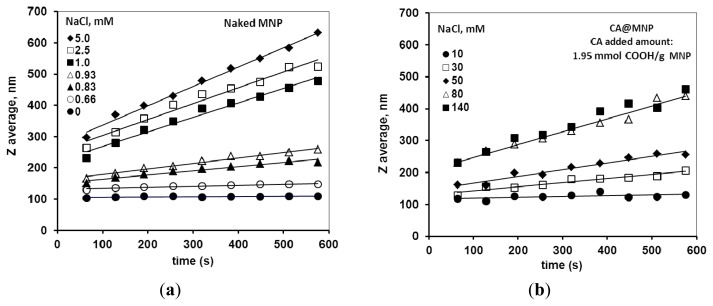
Time dependent increase in the particle size of (**a**) naked; and (**b**) citrate-coated MNPs measured at pH ~6.5 and different NaCl concentrations.

**Figure 7:**
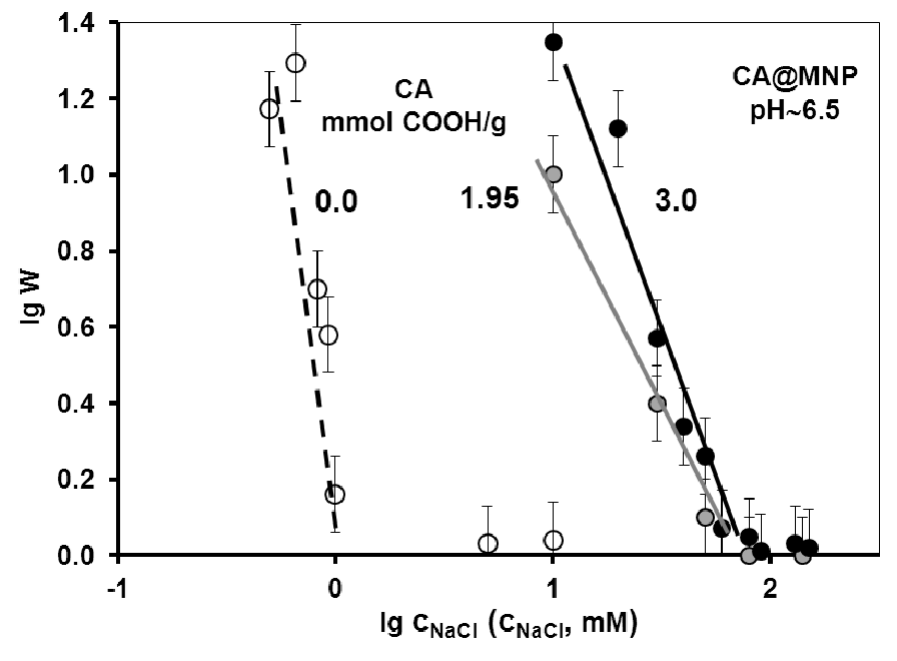
Determination of the CCC values for naked and citrate-coated MNPs at pH ~6.5. The added amounts of CA are 1.95 and 3.0 mmol COOH/g MNP.

The salt tolerance of the CA@MNP formulations was determined in coagulation kinetics experiments at the highest CA contents, since only these dispersions were sufficiently stable in 0.01M NaCl at near physiological pH ~6.5. It is seen in Figure 6a that the hydrodynamic size of the naked MNPs increased with time already in the presence of 1 mM NaCl, while the CA-coated particles (Figure 6b) were fairly stable even at 10 mM NaCl. Significant increase in the particle size of CA@MNPs with time was observed at 80 mM NaCl and above. The plots of the stability constant (W) versus NaCl concentration (Figure 7) allow determining the critical coagulation concentration (CCC) values as ~1 mM for naked MNPs and ~70 mM for CA@MNPs (both for the 1.9 and 3.0 mmol COOH/g MNP samples). The conclusion of these experiments is that CA@MNPs do not stand physiological salt concentration in a simple physicochemical experiment. There are indications in literature that CA@MNPs are also unstable in biological media. For example, Safi *et al.* [49] tested the aggregation kinetics of citrate-coated γ-Fe_2_O_3_ particles in phosphate buffer (PBS) and a cell culture medium (RPMI), and found that the particles underwent rapid coagulation. In addition, the precipitating particles interacted strongly with human lymphoblastoid cells, and a massive and rapid adsorption of iron oxide on the cell surfaces was observed.

The salt tolerance of the carboxylated MNPs increases in the following order: GA (~20 mM) < CA (~70 mM) < PAA (~500 mM) = PAM (~500 mM) as given previously [25,26]. It has also been observed that GA adsorption is able to stabilize the MNPs to the same degree as PAA and PAM when it is allowed to polymerize in the adsorbed layer for longer time. The detailed discussion of the preparation and characterization of GA@MNPs will be the subject of a forthcoming publication. GA-coated MNPs do not qualify for *in vitro* and *in vivo* experiments according to the colloidal stability tests, albeit their performance is outstanding in both adsorption and chemical stability experiments. In conclusion, from the four studied MNPs, only the PAM@MNP is appropriate for further biological testing processes. The present study provides important messages that (i) the surface modifying property of a specific functional group depends greatly on the chemical structure of its host molecule; and (ii) in a consequent physicochemical pre-selection procedure, the biologically adequate core-shell MNPs can be chosen from the whole series of similar candidates. We emphasize that our optimization and selection procedure concerns core-shell formulations based on the physicochemical adsorption of small and large molecular carboxylates on naked iron oxide nanoparticles. Nevertheless, the basic principles discussed here can be adapted in other formulation methods as well.

### 2.4. *In Vitro* and *in Vivo* Performance of Carboxylate@MNPs

We have performed *in vitro* and *in vivo* experiments with the carboxylate@MNPs to test the usefulness and necessity of our selection procedure in identifying physicochemically appropriate nanoparticle formulations. Supposed that the results are consistent with those of cell and animal experiments, this procedure can be used to exclude inadequate formulations from the expensive and time consuming biological testing procedure and clinical trials.

#### 2.4.1. Biocompatibility Tests

All of the CA, GA, PAA and PAM coated nanoparticles have been found nontoxic according to the previous MTT assays [23,24,26]; the viability of both healthy and cancerous cells in their presence decreased by less than 25%, the commonly accepted threshold of cytotoxicity. However, only PAM@MNP is fully adequate for biomedical testing according to the physicochemical tests. This leads to the conclusion that the small differences in the results of the MTT assays should be regarded as signs that differentiate nontoxic nanoparticles from those that are indeed bioapplicable. The latter should remain stable enough in biological media in order to perform their designed function. For example, dissolution of biocompatible core-shell MNPs leads to considerable reduction in saturation magnetization and to weak performance in MRI or magnetic hyperthermia applications [29,48]. The cell inhibition percentage values of the four carboxylated MNPs have been averaged over all types of cells tested and over all concentrations of MNPs (1, 5, 20, and 100 mg/L for CA, GA, PAA, and PAM). The results are shown in Table 2. The cell proliferation inhibition of CA and PAA coated MNPs is significantly higher than that of the GA and PAM coated MNPs in direct correlation with the results of the physicochemical tests. The cell inhibition percentage correlates with the iron-releasing property (dissolved iron), but does not correlate with the colloidal stability of the particles (CCC), meaning that iron dissolution is more important in the aspect of cell viability than nanoparticle aggregation.

**Figure 8:**
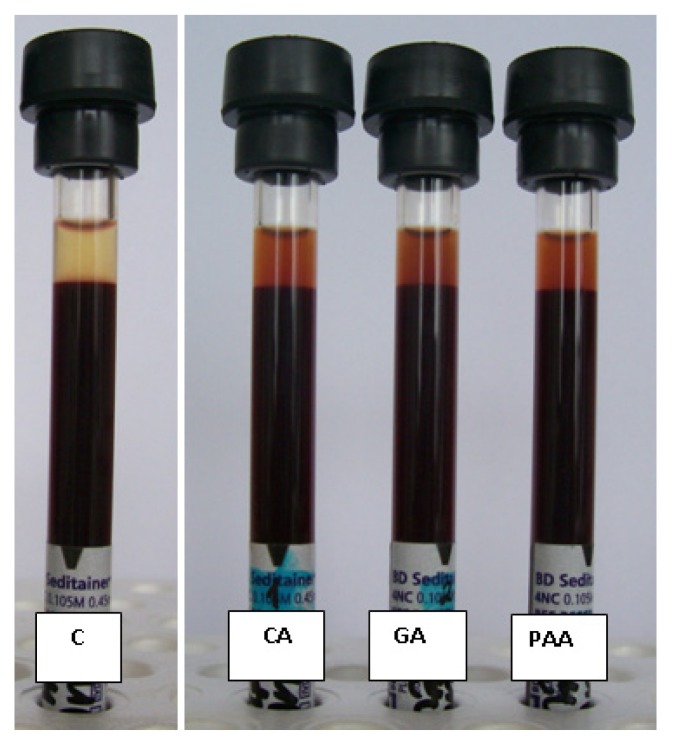
Blood sedimentation experiment: Blood sample of Donor #3 with CA@MNP (CA), GA@MNP (GA) and PAA@MNP (PAA) added at 0.16 mg/mL concentration, in comparison with the control sample (C).

We have examined the erythrocyte sedimentation rate (ESR) of whole blood samples in the presence of CA, GA and PAA coated MNPs added at 0.16 mg/L concentration (Figure 8). Our previous study of the concentration-dependence of ESR in the presence of PAM@MNPs [24] showed no effect of the nanoparticles even at the highest added amount (0.16 mg/L). The same was observed in the present experiments for all the other nanoparticles. It is seen in Figure 8 that the height of sediments was the same both in the presence and absence of the MNPs.

This result could be somewhat surprising in the light of the above discussed variations in the chemical and colloidal stability and nontoxicity level of all particles. It is likely that the protein corona [50,51] adsorbed form the serum can protect them from aggregation. This effect is reflected in the fact that MNP aggregation cannot be seen even in the supernatants of the sedimentation experiments.

**Figure 9:**
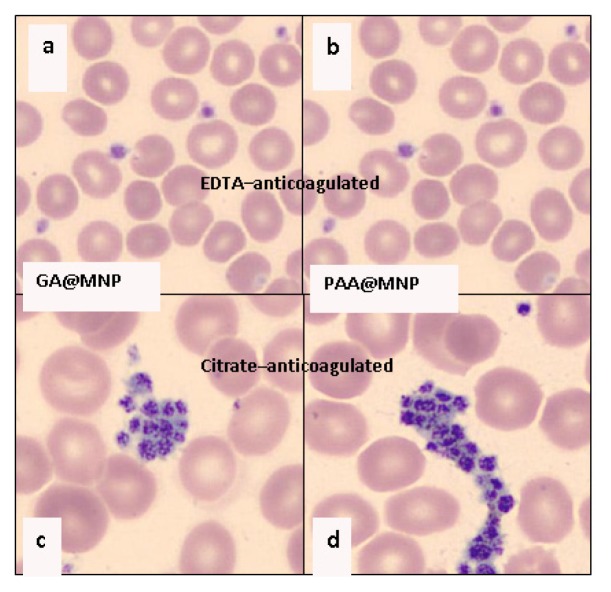
Pictures of the blood smears prepared from (**a**) EDTA-anticoagulated blood with GA@MNP; (**b**) EDTA-anticoagulated blood with PAA@MNP; (**c**) Citrate-anticoagulated blood with GA@MNP; and (**d**) Citrate-anticoagulated blood with PAA@MNP.

We could test the iron dissolution effect of citrate in biological media by performing smear experiments on two series of whole blood samples, one collected in EDTA and the other in Na-citrate coated vacutainer tubes. We have observed thrombocyte aggregation in blood samples with GA or PAA-coated MNPs from citrate-anticoagulated blood, but there were no aggregates in the EDTA-anticoagulated ones (Figure 9). As thrombocyte aggregation is controlled by a very wide variety of biological factors [52], it is difficult to explain why it occurs in citrate-anticoagulated, but not in EDTA-anti-coagulated blood. One of the possible explanations is directly connected with the general observation that surface charge (and surface potential) lowering is always observed in platelet aggregation [53–55]. In the presence of high citrate concentration in the vacutainers (CA/MNP is 0.16 mol/mg) the dissolved Fe-citrate complex can become adsorbed on the platelet surface and decrease the surface potential, while EDTA-chelation prevents iron adsorption. In addition, EDTA itself is known to increase platelet surface charge [55]. According to another observation, thrombocytes can aggregate in the smears from non-anticoagulated blood because of mechanical stress, while EDTA protects thrombocytes from aggregation [56]. In our experiments, however, citrate-anticoagulated control samples without MNPs did not show the signs of thrombocyte aggregation. It is apparent that the displacement of the coating shells of MNPs by the large amount of citrate causes iron dissolution and thrombocyte aggregation.

#### 2.4.2. Chemical Stability of Carboxylate@MNPs in Biological Media

**Figure 10:**
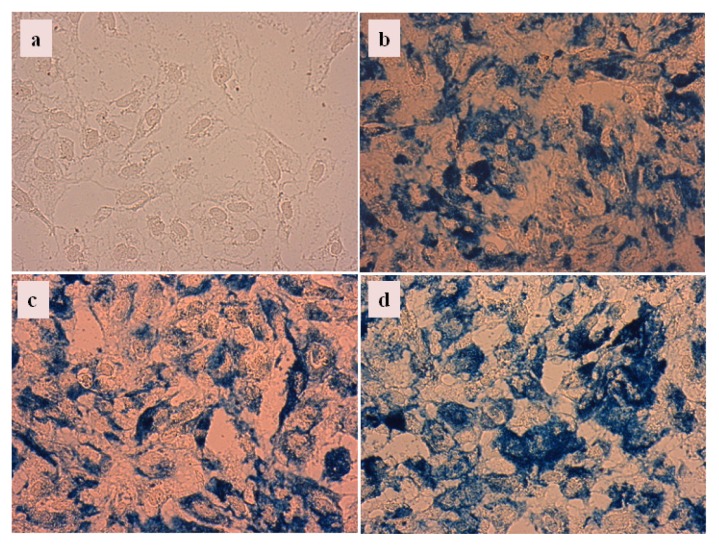
Prussian blue staining of HeLa cells (**a**) incubated with 14.7 mg/mL concentration dispersions of CA@MNP (**b**); PAA@MNP (**c**); and PAM@MNP (**d**).

We have chosen the CA, PAA, and PAM-coated MNPs to test iron dissolution in biological media. *In vitro* experiments were performed in HeLa cell cultures prepared the same way as in the MTT tests. Prussian blue staining was applied after the second day of growing to visualize the distribution of iron. It is seen in Figure 10 that stained iron is localized in high concentration near the HeLa cells and the light blue colored background indicates the presence of iron at very low concentration. In the case of CA@MNPs (Figure 10b) the extended light blue colored areas spread across the intercellular space in a diffuse manner, indicating iron leaching from the MNPs. Iron leaching is less significant in the PAA@MNP (Figure 10c) and practically absent in the PAM@MNP (Figure 10d) incubated cell cultures; the contour of the blue-stained spots becomes increasingly sharper from CA to PAA to PAM coating. These somewhat subjective, but significant observations correlate with the iron dissolution results of the physicochemical experiments and support that only PAM@MNPs are efficiently passivized.

**Figure 11:**
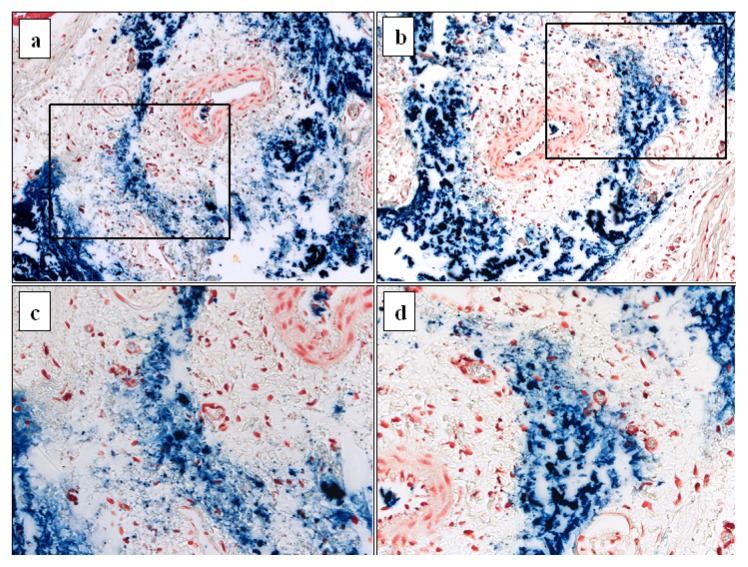
Prussian blue staining of rat tail tissues with IV injected carboxylate@MNP particles: (**a**) CA@MNP at 20x; and (**b**) PAA@MNP at 20x magnification; (**c**) and (**d**) are the 40× magnification of the selected areas of (**a**) and (**b**).

In *in vivo* experiments, the CA@MNP and PAA@MNP dispersions were injected intravenously in rat tails. The iron leaching feature was observed (Figure 11), similarly to that in cell cultures. The veins apparently crashed upon injection and the majority of nanoparticles spread into the tissues around the veins. However, nanoparticles have also been transported by the vascular system; blue-stained particles are present in the arteries (white areas surrounded by the array of wall cells) as well.

The light blue colored hazy background of low iron concentration is spilled into the intercellular space much more diffusely and extensively in the CA@MNP injected tissue than in the PAA@MNP treated one. At the present state of studies, it cannot be known, whether Prussian blue staining reflects the presence of dissolved iron in the form of citrate (or biomolecular) complexes indeed. Similar pictures are frequently seen in literature [57–61], but the main concern in general is the iron-positivity and not the exact form of the iron. On the other hand, the above observations closely correlate with the iron dissolution results in simple aqueous media and so, iron dissolution in the biological media is also probable. In addition, *in vivo* iron dissolution from citrate-coated nanoparticles is found by other authors as well [28,29].

#### 2.4.3. Cellular Uptake of Carboxylate@MNPs

**Figure 12:**
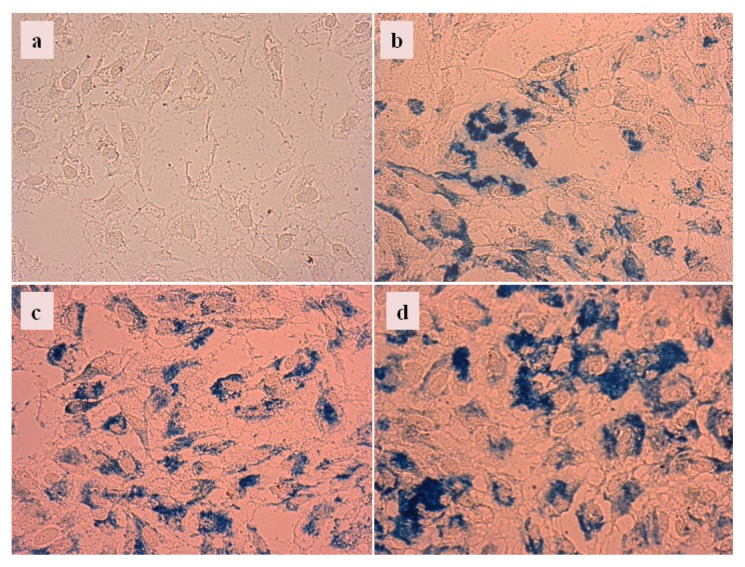
Prussian blue staining of HeLa cells (**a**) incubated with 7.35 mg/mL concentration dispersions of CA@MNP (**b**); PAA@MNP (**c**); and PAM@MNP (**d**).

We have incubated the HeLa cell cultures with CA, PAA and PAM-coated nanoparticles at low concentration, namely, half of that in iron dissolution tests (Figure 10), for tracking cell internalization of MNPs. The latter is seen as accumulation of MNPs within the cells around the nucleus. The cellular interactions of these MNPs were significantly different: CA@MNPs were both internalized and adsorbed (Figure 12b), while PAA@MNPs preferably internalized (Figure 12c) and PAM@MNP mainly adsorbed on the cell surface (Figure 12d).

This finding is very important. It supports one of the conclusions from physicochemical characterizations that despite all the MNPs are covered with carboxylated shell; their fate in the cellular environment can be very much different depending on the chemistry of carboxylates and the compactness of shell. It is known that negatively charged nanoparticles are prone to cell internalization [21], which is evidently not the case after 48 h incubation in our tests with PAM@MNPs and CA@MNPs.

## 3. Experimental Section

### 3.1. Materials

Magnetite (Fe_3_O_4_) nanoparticles (MNPs) were synthesized from the mixture of FeCl_2_ and FeCl_3_ salts, according to the alkaline hydrolysis method [62–65]. The size of the resulting particles was determined based on transmission electron microscopic pictures as d ~8–10 nm (d is the mean particle diameter). Citric acid (CA), gallic acid (GA), poly(acrylic acid) (PAA, *M*_w_ = 1800 Da) and poly(acrylic acid-*co*-maleic acid) (PAM, *M*_w_ = 3000 Da, 50 *wt.* % in H_2_O) were purchased from Sigma-Aldrich. The concentrations of the carboxylic acids are expressed through the molar amount of carboxylic groups. In the case of PAA and PAM the carboxyls in the monomeric units are taken into account: –COOH/*M*_w,AA_ = 1/72 = 0.0139 mol/g and –COOH/*M*_w,AM_ = 3/188 = 0.0159 mol/g, *i.e.*, 1 g of PAA (or PAM) contains 0.0139 (or 0.0159) mol COOH. The pH and ionic strength were set by solutions of NaCl, HCl and NaOH, analytical grade products of Molar (Hungary). Milli-Q water was used. All experiments were performed at room temperature (25 ± 1 °C).

**Figure 13:**
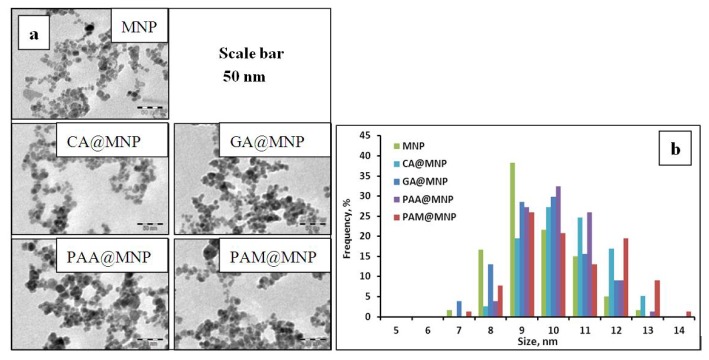
Transmission electron micrographs of the MNP, CA@MNP, GA@MNP, PAA@MNP and PAM@MNP samples (**a**); and their size distribution (**b**).

TEM micrographs of the naked and the CA-, GA-, PAA- and PAM-coated MNPs, taken using a Philips CM-10 transmission electron microscope supplied with a Megaview-II camera, are presented in Figure 13a. The accelerating voltage was 100 kV and the maximum resolution of the instrument is 0.2 nm. The particles were deposited on Formwar-coated copper grids from highly diluted suspensions and the samples were dried under infrared light. The size distribution (Figure 13b) was 9.7 ± 1.0, 10.5 ± 1.4, 9.4 ± 0.8, 10.1 ± 0.9 and 10.8 ± 1.2 nm for the naked MNP, CA@MNP, GA@MNP, PAA@MNP and PAM@MNP, respectively, as determined by evaluating 100 particles using the UTHSCSA Image Tool 2.00 software.

HRTEM pictures and XRD spectra of the naked MNPs have been published [66] and showed a crystalline structure with well resolved magnetite diffraction peaks.

### 3.2. Methods

The adsorption and desorption isotherms of the polyacids were determined by batch method at pH = 6.5 ± 0.5 (denoted in the text as pH ~6.5) and constant ionic strength, 0.01 M (set by NaCl). Magnetite suspensions were equilibrated with the series of polyacid solutions in closed test tubes for 24 h at room temperature. The suspension concentrations were 1–20 g/L and the highest polyacid concentration was 10 mmol/L. The pH was adjusted by NaOH or HCl solution, and checked at the end of the adsorption time. The equilibrium concentrations of GA, PAA and PAM were determined spectrophotometrically in a USB4000 spectrometer (Ocean Optics, Dunedin, FL, USA) and those of CA by cerimetric titration using ferroin indicator. The absorbance of the supernatants at 260 nm (GA) or the difference in the absorbances at 223 and 250 nm (both PAA and PAM) were determined after perfect separation of the solid particles by centrifuging at 13,000 RPM for one hour. At higher polyacid concentrations, the separation was assisted by a permanent magnet and membrane filtration (0.22 μm MILLEX-GP). The iron concentration was determined in the supernatants of selected samples along the adsorption isotherms: four of them evenly distributed in the rising part and two of them in the plateau region. The iron content was measured in an Agilent 7700x ICP-MS spectrometer. Three parallel determinations were performed for each sample, and the measurement error was ±25 μg/L. Below the threshold of ~200 ppb, reliable Fe concentration cannot be determined. The separated sediments were next redispersed in carboxylate solutions for measuring desorption isotherms. The concentration of the carboxylates was half of that in the adsorption. The equilibrium concentration was determined as in the adsorption experiments. The value of *SD*_(_*_n_*_−1)_ = 0.12 was calculated for the adsorption and desorption experiments.

FTIR-ATR spectra were recorded with a Bio-Rad Digilab Division FTS-65A/896 spectrometer (with DTGS detector), using a Harrick’s Meridian Split Pea Diamond ATR accessory. The absorbance of the samples was measured in single reflection mode in the 400–4000 cm^−1^ frequency range with resolution of 2 cm^−1^, accumulating 256 scans. Magnetite suspensions, polyacid solutions or suspensions of the polyacid-coated MNPs were dried on the crystal surface.

Electrophoretic mobilities of the pure (naked) and the polyacid-coated magnetite samples were measured in a NanoZS (Malvern, UK) apparatus at 25 ± 0.1 °C, in disposable zeta cells (DTS 1060). The settings of the instrument were checked by using a standard latex sample, the zeta potential of which is reported as ~(55 ± 5) mV by Malvern. The optimal scattering condition (~10^5^ counts per seconds) was set by either 0.05 or 0.1 g/L magnetite concentration depending on the aggregation state of the dispersions. The pH was changed between ~3 and ~10 keeping the added amount of polyacids (per one gram of MNP) constant, or alternatively, the added amount of polyacids was varied at pH ~6. The ionic strength was constant: 0.005 M (CA) and 0.01 M (GA, PAA and PAM). The measurements were started after one hour equilibration time followed by an ultrasonic agitation for 10 s. The standard deviation of measured data was *SD*_(_*_n_*_− 1)_ = 1.

The hydrodynamic diameter of the particles was measured in a NanoZS apparatus (Malvern, UK) with a He-Ne laser (λ = 633 nm), operating in backscattering mode at an angle of 173°. The stock sols of magnetite particles were diluted with NaCl electrolyte to 0.1 g/L solid content. The ionic strength was constant as in the electrokinetic potential measurements and pH was adjusted in the range of 3 to 10, directly before the measurements. Before all measurements, the samples were homogenized by ultrasound agitation for 10 s and allowed to relax for 110 s. The average values of the hydrodynamic diameter (*Z*_average_) were calculated from the 3rd order cumulant fits of the correlation functions. The values of standard deviation of *Z*_average_ varied between 15 (for the primary particles) and 150 (for aggregates of the size of ~1500 nm).

Salt tolerance of the carboxylate@MNPs was tested in coagulation kinetics experiments at pH ~6.5 by using a Zetasizer 4 (Malvern, UK) apparatus. The MNP concentration was set to 0.0025 g/L in order to achieve the optimum of light scattering and particle diffusion, and the aggregation was followed by detecting the evolution of the particle size (*Z*_average_) up to 600 s. The coagulation rate was calculated from the slope of the kinetic curves. The stability ratio (*W*) was calculated from the initial slopes of kinetic curves belonging to the slow and fast coagulation ranges. The critical coagulation concentration (CCC) was determined from the log_10_*W vs.* log_10_*C*_NaCl_ (NaCl concentration) function. The standard deviation of the values of *W* was *SD*_(_*_n_*_− 1)_ = 0.2.

The magnetization curves were measured in a vibrating sample magnetometer VSM 880 (DMS/ADE Technologies-USA) at the NCESCF-UP Timisoara. The analysis was performed on the carboxylate@MNP dispersions at ~10% by weight up to 10000 Oe as the maximum of the applied field. The values of specific magnetization were calculated for the exact amount of pure MNP in the samples.

The interaction of CA, PAA and PAM coated MNPs with HeLa cells isolated from human cervix adenocarcinoma (ECACC, Salisbury, UK) was evaluated by the Prussian blue staining method. The cells were cultivated in minimal essential medium supplemented with 10% fetal bovine serum, 1% non-essential amino acids and an antibiotic-antimycotic mixture. All media and supplements were obtained from PAA Laboratories GmbH (Pasching, Austria). Near-confluent cancer cells were seeded onto a 96-well micro-plate at the density of 5000 cells/well. After an overnight standing, a 200 μL aliquot of the above medium containing the carboxylate@MNPs at 14.7 and 7.35 mg/mL concentration was added. After incubation for 48 h at 37 °C in humidified air containing 5% CO_2_, ferric ion content of the cells was visualized by Prussian blue staining. The medium was removed and a 1:1 mixture of 2% potassium ferrocyanide and 2% hydrochloric acid was added for 10 min. Then the wells were washed with phosphate-buffered saline and the cells were photographed by means of a Nikon Eclipse microscope equipped with a QCapture CCD camera.

Blood sedimentation in the presence of carboxylate@MNPs was tested in erythrocyte sedimentation rate (ESR) experiments, using a Sedi-15 automated blood sedimentation instrument (BD Inc., Franklin Lakes, NJ, USA) and Seditainer 1.8 vacutainer tubes (BD Inc., USA). The CA, GA or PAA coated particles (with carboxylate concentrations of 0.21, 0.61 and 1.02 mmol/g MNP, respectively) were added to citrate-anticoagulated whole blood of three healthy volunteers at a concentration of 0.16 mg/L. The ESR values were determined after 25 min standing and the accuracy of the measurements was ±3 mm/h.

Blood smears were prepared by an automated slide preparation system (Sysmex SP4000i) using the May-Grünwald Giemsa (MGG, Biolyon, Dardilly, France) staining technique. For analysis of the smears an automated CellaVisionTM DM96 device (Cellavision AB, Ideon, Science Park, Lund, Sweden) was used, comprising a slide feeder unit, a microscope with ×10, ×50, and ×100 objectives, a digital camera, a computer, and an acquisition and classification software. The software classifies captured cells into 12 leukocyte categories, 6 RBC categories (normal and abnormal), aggregated thrombocytes, artifacts and smudged cells. GA@MNP and PAA@MNP dispersions were added to the citrate- and the EDTA-anticoagulated whole blood samples of Donor #3 at a concentration of 0.16 mg/L, as above. The samples were homogenized, allowed to stand at room temperature for 10 min, and homogenized again prior to smear preparation.

*In vivo* testing of the carboxylated MNPs was performed by IV injection of 100 μL of the MNP dispersions into the tails of 6 Wistar rats. The dispersions were prepared at a concentration of 10 mg/mL carboxylate@MNPs (using the optimal stabilizing amounts of the carboxylates) in physiological salt solution. Tissue samples were taken for examination at 1 h after the injection and stored in formaldehyde overnight. For optical microscopy, the tissue segments were embedded in paraffin and stained with combined Hematoxylin and Eosin. Prussian blue staining was used for iron detection.

## 4. Conclusions

We have shown that a designed physicochemical testing of magnetite core-shell nanoparticles for biomedical applications is an effective tool to exclude a priori inadequate formulations from *in vitro* and *in vivo* tests. The selection procedure should comprise (1) qualitative and quantitative characterization of shell formation on magnetic core (isotherm measurement); (2) determination of interaction strength between coating molecules and MNP surface; (3) quantitative optimization of coating to stabilize MNPs efficiently; (4) characterization of core-passivizing efficiency; (5) determination of particle charge (electrokinetic potential measurements); and (6) salt tolerance measurement of core-shell MNPs. The previously published PAA, PAM and GA-coated MNPs have been chosen to demonstrate the selection process. Its validity has been shown through a series of new experiments for CA-coated MNP as well. Although citric acid is a very popular coating molecule, CA@MNPs become entirely disqualified in the course of our selection procedure. This can explain to some extent the failure of such types of formulations in clinical trials, official (FDA, EMA or equivalent) approval or production/marketing to date.

**Figure 14:**
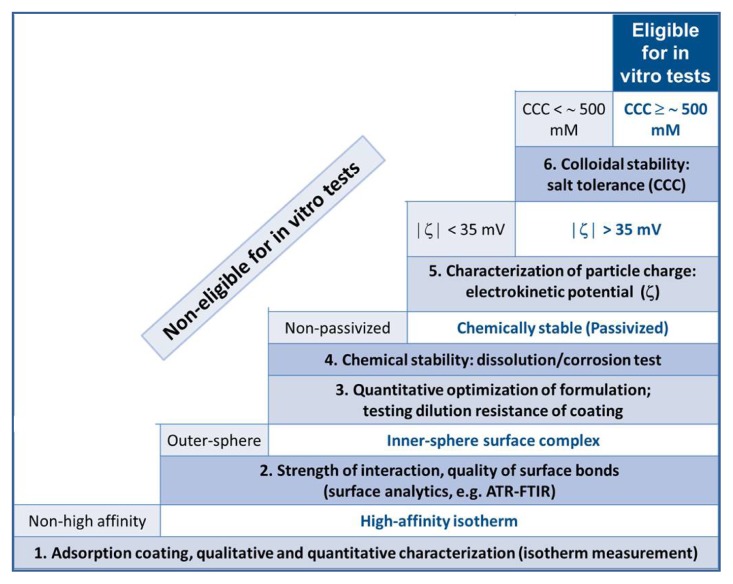
Schematic presentation of the suggested optimization procedure of carboxylate@MNP core-shell nanoparticles developed for biomedical applications; the route of preparation is the adsorption of the carboxylates to the naked MNPs.

For the sake of easy overview, we presented the scheme in Figure 14 that we have applied in the specific case of core-shell magnetite nanoparticles preparation based on adsorption of small and macromolecular carboxylates. The sequence of the experiments is important, since the basic properties such as the efficiency of core-shell fastening (strength of interaction), compactness of shell (passivizing efficiency), *etc.*, determine the final behavior of the nano-composites and only the particles with the best coating quality should be tested for colloidal stability. In addition, all physicochemical tests must precede the *in vitro* and *in vivo* experiments to prove that the minimum criteria of biological applicability (*i.e*., the chemical and colloidal stability of the formulations at biological pH and NaCl concentrations) are fulfilled.

It is known from the literature that protein corona formation can in principle hinder aggregation in physiological media, even if the colloidal stability tests predict aggregation. Thus, the next step in the testing of MNPs should be the *in vitro* study of protein adsorption. However, the coating shell of the particles with the highest colloidal stability may provide a better anchoring space for proteins in comparison with loosely packed shells. The importance of the chemical stability of the core-shell MNPs cannot be overestimated. It is highly advisable that chemically labile MNPs (with measurable corrosion or dissolution) are not allowed for *in vitro* experiments. We have experienced the obvious dissolution of iron from CA@MNPs and PAA@MNPs in optical imaging of cell cultures and the other *in vitro* and *in vivo* tests such as blood smears and tissue sections, while there was no sign of it in MTT tests. Iron dissolution enhances the toxicity of carboxylate@MNPs through oxidative stress and lowers the magnetic response because of a decrease in the magnetic core size.

## Figures and Tables

**Table 1:** The amounts of added COOH groups needed to reach characteristic conditions (*i.e*., high-affinity adsorption limit, IEP and overcharging) in the carboxylate@MNP dispersions at pH ~6.5 and *I* = 0.01 M.

Characteristic points	CA	GA	PAA	PAM

	mmol COOH/g MNP			
High-affinity adsorption limit	0.33	0.17	-	0.32
Isoelectric point (charge neutralization)	0.45	0.05	0.13	0.17
Complete overcharging	~0.8	0.4	0.4	0.4

**Table 2:** Correlation between the averaged results of MTT assays [23,24,26] and the results of physicochemical measurements (iron dissolution and coagulation kinetics experiments).

Carboxylate coating	Averaged inhibition %	Dissolved iron mg/g MNP	CCC mM
CA	18.3 ± 1.7	1.276	70
GA	1.5 ± 0.5	0	20
PAA	12.5 ± 1.4	0.038	500
PAM	1.5 ± 0.5	0	500
